# Supplementary material for: Are behavioral economics interventions effective in increasing colorectal cancer screening uptake: A systematic review of evidence and meta-analysis?
Source: PLoS One. 2024 Feb 5;19(2):e0290424. doi: 10.1371/journal.pone.0290424 (PMC10843112; doi:10.1371/journal.pone.0290424)
Supplement: S1 Appendix — (DOCX) [file pone.0290424.s003.docx]

**Appendix**

Table A: Search strategies

| **Data base** | **Search strategy** | **#** |
| --- | --- | --- |
| PubMed | Search1: ((Bowel cancer OR colorectal cancer OR faecal immunochemical test* OR faecal occult blood test* OR colonoscopy OR flexible sigmoidoscopy OR colonography) AND (screen*) AND (behavioural economics OR behavioral economics OR nudg* OR messenger OR incentiv* OR norms OR default* OR salience OR priming OR commitment) AND (behaviour OR behavior OR participation OR adherence OR uptake) AND (randomised controlled trial OR randomized controlled trial OR field experiment OR randomised trial OR randomized trial OR RCT OR controlled trial)) Filters: Abstract, Free full text, Full text, Clinical Trial, Meta-Analysis, Randomized Controlled Trial, Review, Systematic Review, from 2000 – 2022 | 93 |
|  | Search2: ((Bowel cancer OR colorectal cancer OR faecal immunochemical test* OR faecal occult blood test* OR colonoscopy OR flexible sigmoidoscopy OR colonography OR FIT OR FOBT OR gFOBT OR FS) AND (screen*) AND (behavioural economics OR behavioral economics OR nudg* OR messenger OR incentiv* OR norms OR default* OR salience OR priming OR heuristics OR bias OR aversion OR decision fatigue OR regret) AND (behaviour OR behavior OR participation OR adherence OR uptake OR utilisation OR utilization) AND (randomised controlled trial OR randomized controlled trial OR field experiment OR randomised trial OR randomized trial OR RCT OR controlled trial)) Filters: Abstract, Free full text, Full text, Clinical Trial, Meta-Analysis, Randomized Controlled Trial, Review, Systematic Review, English, from 2000 - 2022 | 236 |
|  | Search3: ((Bowel cancer OR colorectal cancer OR faecal immunochemical test* OR faecal occult blood test* OR colonoscopy OR flexible sigmoidoscopy OR colonography OR FIT OR FOBT OR gFOBT OR FS OR colon cancer OR rectal cancer) AND (screen*) AND (behavioural economics OR behavioral economics OR nudg* OR messenger OR incentiv* OR norms OR default* OR salience OR priming OR commitment OR heuristics OR bias OR aversion OR decision fatigue OR regret OR order effect*) AND (behaviour OR behavior OR participation OR adherence OR uptake OR utilisation OR utilization OR practices) AND (randomised controlled trial OR randomized controlled trial OR field experiment OR randomised trial OR randomized trial OR RCT OR controlled trial)) Filters: Abstract, Free full text, Full text, Clinical Trial, Meta-Analysis, Randomized Controlled Trial, Review, Systematic Review, English, from 2000 - 2022 | 280 |
| Scopus | ALL((Bowel cancer OR colorectal cancer OR faecal immunochemical test* OR faecal occult blood test* OR colonoscopy OR flexible sigmoidoscopy OR colonography) AND (screen*) AND (behavioural economics OR behavioral economics OR nudg* OR incentiv* OR norms OR default* OR salience OR priming OR commitment) AND (behaviour OR behavior OR participation OR adherence OR uptake) AND (randomised controlled trial OR randomized controlled trial OR field experiment OR randomised trial OR randomized trial OR RCT OR controlled trial)) AND (exclude (pubyear,1997)) AND (exclude(subjarea,"bioc") OR exclude (subjarea,"ceng") OR exclude (subjarea,"chem") OR exclude (subjarea,"engi") OR exclude (subjarea,"immu")) AND (limit-to (doctype,"ar") OR limit-to (doctype,"re") OR limit-to (doctype,"cp")) AND (limit-to( language,"english")) | 56 |
|  | ALL((Bowel cancer OR colorectal cancer OR faecal immunochemical test* OR faecal occult blood test* OR colonoscopy OR flexible sigmoidoscopy OR colonography OR FIT OR FOBT OR gFOBT OR FS) AND (screen*) AND (behavioural economics OR behavioral economics OR nudg* OR incentiv* OR norms OR default* OR salience OR priming OR commitment OR heuristics OR bias OR aversion OR decision fatigue OR regret) AND (behaviour OR behavior OR participation OR adherence OR uptake OR utilisation OR utilization) AND (randomised controlled trial OR randomized controlled trial OR field experiment OR randomised trial OR randomized trial OR RCT OR controlled trial)) AND (limit-to ( pubyear,2022) OR limit-to (pubyear,2021) OR limit-to (pubyear,2020) OR limit-to (pubyear,2019) OR limit-to (pubyear,2018) OR limit-to (pubyear,2016) OR limit-to (pubyear,2013) OR limit-to (pubyear,2006)) AND (exclude (subjarea,"bioc")) AND (limit-to (doctype,"ar") OR limit-to ( doctype,"re")) AND (limit-to (language,"english")) | 8 |
|  | ALL((Bowel cancer OR colorectal cancer OR faecal immunochemical test* OR faecal occult blood test* OR colonoscopy OR flexible sigmoidoscopy OR colonography OR FIT OR FOBT OR gFOBT OR FS OR colon cancer OR rectal cancer) AND (screen*) AND (behavioural economics OR behavioral economics OR nudg* OR incentiv* OR norms OR default* OR salience OR priming OR commitment OR heuristics OR bias OR aversion OR decision fatigue OR regret OR order effect*) AND (behaviour OR behavior OR participation OR adherence OR uptake OR utilisation OR utilization OR practices) AND (randomised controlled trial OR randomized controlled trial OR field experiment OR randomised trial OR randomized trial OR RCT OR controlled trial)) AND (limit-to (pubyear,2022) OR limit-to (pubyear,2021) OR limit-to (pubyear,2020) OR limit-to (pubyear,2019) OR limit-to (pubyear,2018) OR limit-to (pubyear,2017) OR limit-to (pubyear,2016) OR limit-to (pubyear,2015) OR limit-to (pubyear,2013) OR limit-to (pubyear,2012) OR limit-to (pubyear,2008) OR limit-to (pubyear,2007) OR limit-to (pubyear,2006)) AND (exclude (subjarea,"bioc") | 16 |
| Cochran | ((Bowel cancer OR colorectal cancer OR faecal immunochemical test* OR faecal occult blood test* OR colonoscopy OR flexible sigmoidoscopy OR colonography) AND (screen*) AND (behavioural economics OR behavioral economics OR nudg* OR incentiv* OR norms OR default* OR salience OR priming OR commitment) AND (behaviour OR behavior OR participation OR adherence OR uptake) AND (randomised controlled trial OR randomized controlled trial OR field experiment OR randomised trial OR randomized trial OR RCT OR controlled trial)) in Title Abstract Keyword - with Cochrane Library publication date Between Jan 2000 and Apr 2022, in Trials (Word variations have been searched) | 195 |
|  | Trials matching ((Bowel cancer OR colorectal cancer OR faecal immunochemical test* OR faecal occult blood test* OR colonoscopy OR flexible sigmoidoscopy OR colonography OR FIT OR FOBT OR gFOBT OR FS) AND (screen*) AND (behavioural economics OR behavioral economics OR nudg* OR incentiv* OR norms OR default* OR salience OR priming OR commitment OR heuristics OR bias OR aversion OR decision fatigue OR regret) AND (behaviour OR behavior OR participation OR adherence OR uptake OR utilisation OR utilization) AND (randomised controlled trial OR randomized controlled trial OR field experiment OR randomised trial OR randomized trial OR RCT OR controlled trial)) in Title Abstract Keyword - with Cochrane Library publication date Between Jan 2000 and Apr 2022, in Trials (Word variations have been searched) | 451 |
|  | Trials matching ((Bowel cancer OR colorectal cancer OR faecal immunochemical test* OR faecal occult blood test* OR colonoscopy OR flexible sigmoidoscopy OR colonography OR FIT OR FOBT OR gFOBT OR FS OR colon cancer OR rectal cancer) AND (screen*) AND (behavioural economics OR behavioral economics OR nudg* OR incentiv* OR norms OR default* OR salience OR priming OR commitment OR heuristics OR bias OR aversion OR decision fatigue OR regret OR order effect*) AND (behaviour OR behavior OR participation OR adherence OR uptake OR utilisation OR utilization OR practices) AND (randomised controlled trial OR randomized controlled trial OR field experiment OR randomised trial OR randomized trial OR RCT OR controlled trial)) in Title Abstract Keyword - with Cochrane Library publication date Between Jan 2000 and Apr 2022, in Trials (Word variations have been searched) | 631 |

Table B. Quality assessment table (The Modified Jadad Scale)

| **Study** | **Was the research described as randomized?** | **Was the approach of randomization appropriate?** | **Was the research described as blinding?** | **Was the approach of blinding appropriate?** | **Was there a presentation of withdrawals and dropouts?** | **Was there a presentation of the inclusion/exclusion criteria?** | **Was the approach used to assess adverse effects described?** | **Was the approach of statistical analysis described?** | **Total** |
| --- | --- | --- | --- | --- | --- | --- | --- | --- | --- |
| Jane Wardle et al | 1 | 1 | 0 | 0 | 0 | 1 | 0 | 1 | 4 |
| Isaac M. Lipkus et al | 1 | 1 | 0 | 0 | 0 | 1 | 0 | 1 | 4 |
| Ronald E. Myers et al 2014 | 1 | 1 | 0 | 0 | 1 | 1 | 0 | 1 | 5 |
| Lyndal J Trevena et al | 1 | 1 | 1 | 1 | 1 | 1 | 0 | 1 | 7 |
| Anke Steckelberg et al | 1 | 1 | 1 | 1 | 1 | 1 | 0 | 1 | 7 |
| David P. Miller et al 2011 | 1 | 1 | 0 | 0 | 0 | 1 | 0 | 1 | 4 |
| Michael Pignone et al | 1 | 1 | 0 | 0 | 0 | 1 | 0 | 1 | 4 |
| Sally W. Vernon et al | 1 | 1 | 1 | 1 | 1 | 1 | 0 | 1 | 7 |
| Usha Menon et al | 1 | 1 | 0 | 0 | 0 | 1 | 0 | 1 | 4 |
| Ronald E. Myers et al 2007 | 1 | 0 | 1 | 0 | 1 | 1 | 0 | 1 | 5 |
| Siu Hing Lo et al | 1 | 1 | 0 | 0 | 1 | 0 | 0 | 1 | 4 |
| Efrat Neter et al | 1 | 1 | 0 | 0 | 0 | 1 | 0 | 1 | 4 |
| Jeffrey T. Kullgren et al 2014 | 1 | 1 | 1 | 1 | 1 | 1 | 0 | 1 | 7 |
| Kathleen Clouston et al | 1 | 1 | 1 | -1 | 0 | 1 | 0 | 1 | 4 |
| Paul C. Schroy et al | 1 | 1 | 0 | 0 | 1 | 1 | 0 | 1 | 5 |
| Ronald E. Myers et al 2013 | 1 | 0 | 1 | 1 | 1 | 1 | 0 | 1 | 6 |
| Ronan E. O’Carroll et al | 1 | 1 | 1 | 0 | 1 | 1 | 0 | 1 | 6 |
| Alison T. Brenner et al | 1 | 1 | 0 | 0 | 1 | 1 | 0 | 1 | 5 |
| Schwartz et al | 1 | 1 | 0 | 0 | 1 | 1 | 0 | 1 | 5 |
| Shivan J. Mehta et al 2017 | 1 | 1 | 1 | 1 | 0 | 1 | 0 | 1 | 6 |
| David P. Miller et al 2018 | 1 | 1 | 1 | 1 | 0 | 1 | 0 | 1 | 6 |
| Mehta et al | 1 | 1 | 1 | 1 | 0 | 1 | 0 | 1 | 6 |
| Beverly B. Green et al | 1 | 1 | 1 | 0 | 0 | 1 | 0 | 1 | 5 |
| Pernille Gabel et al | 1 | 1 | 0 | 0 | 0 | 1 | 0 | 1 | 4 |
| S. Stoffel et al 2019 | 1 | 1 | 0 | 0 | 0 | 1 | 0 | 1 | 4 |
| Shivan J. Mehta and Vikranth Induru et al 2019 | 1 | 1 | 0 | 0 | 0 | 1 | 0 | 1 | 4 |
| Shivan J. Mehta and Rebecca S. Pepe et al 2019 | 1 | 1 | 1 | 1 | 0 | 1 | 0 | 1 | 6 |
| Omar Bakr et al | 1 | 1 | 0 | 0 | 1 | 1 | 0 | 1 | 5 |
| Shivan J. Mehta and Akinbowale Oyalowo et al 2020 | 1 | 1 | 1 | 1 | 0 | 1 | 0 | 1 | 6 |
| Shivan J. Mehta and Catherine Reitz et al 2020 | 1 | 1 | 1 | 1 | 0 | 1 | 0 | 1 | 6 |
| Sandro Stoffel et al | 1 | 1 | 1 | 1 | 1 | 1 | 0 | 1 | 7 |
